# Drug resistance characteristics of *Mycobacterium tuberculosis* isolates obtained between 2018 and 2020 in Sichuan, China

**DOI:** 10.1017/S0950268822000127

**Published:** 2022-01-28

**Authors:** Ruifeng Zhou, Tianli Zheng, Dongxia Luo, Ma Zhu, Qingfeng Li, Yuanhong Xu, Dongmei Wang, Jia Luo, Chongliang Zeng, Guo Wei, Mingyuan Tang, Xing Zhao, Mi Zhou, Haojiang Zuo, Xiaofang Pei

**Affiliations:** 1 Department of Clinical Laboratory, Chengdu Public Health Clinical Centre, Chengdu 610016, Sichuan Province, PR China; 2 West China School of Public Health/West China Fourth Hospital, Sichuan University, Chengdu 610041, Sichuan Province, PR China; 3 Food Safety Monitoring and Risk Assessment Key Laboratory of Sichuan Province, Chengdu 610041, China

**Keywords:** Drug resistance, GeneXpert MTB/RIF assay, multidrug-resistant tuberculosis (MDR-TB), *Mycobacterium tuberculosis*

## Abstract

We investigated the drug resistance of *Mycobacterium tuberculosis* isolates from patients with tuberculosis (TB) and HIV, and those diagnosed with only TB in Sichuan, China. TB isolates were obtained from January 2018 to December 2020 and subjected to drug susceptibility testing (DST) to 11 anti-TB drugs and to GeneXpert MTB/RIF testing. The overall proportion of drug-resistant TB (DR-TB) isolates was 32.1% (*n* = 10 946). HIV testing was not universally available for outpatient TB cases, only 29.5% (3227/10 946) cases had HIV testing results. The observed proportion of multidrug-resistant TB (MDR-TB) isolates was almost double than that of the national level, with approximately 1.5% and 0.1% of the isolates being extensively drug resistant and universally drug resistant, respectively. The proportions of resistant isolates were generally higher in 2018 and 2019 than in 2020. Furthermore, the sensitivities of GeneXpert during 2018–2020 demonstrated a downward trend (80.9, 95% confidence intervals (CI) 76.8–85.0; 80.2, 95% CI 76.4–84.1 and 75.4, 95% CI 70.7–80.2, respectively). Approximately 69.0% (7557/10 946) of the TB cases with DST results were subjected to GeneXpert detection. Overall, the DR-TB status and the use of GeneXpert in Sichuan have improved, but DR-TB challenges remain. HIV testing for all TB cases is recommended.

## Introduction

Drug-resistant tuberculosis (DR-TB) remains a global issue and serious public health challenge. In 2019, the incidence of rifampicin-resistant TB was approximately 500 000 worldwide, with 78% of these cases being multidrug-resistant TB (MDR-TB) [1]. China ranked third globally, after India and Indonesia, in the number of TB cases in 2019 and also has the second highest global MDR-TB burden, accounting for 14% of the burden [1]. Furthermore, the prevalence of MDR-TB in this country varies regionally [2], at 13.3% in Sichuan [3], 9% in Beijing [4], 5% in Shanghai [5].

Sichuan is one of the most important provinces in Southwest China [6]. It comprises 21 regions and 183 counties, with a total area of 486 000 square kilometres. In 2020, the province had a recorded population of 83 million, ranked sixth (RMB 4859.88 billion) in total GDP and ranked first with an economic growth rate of 3.8% in China. Additionally, Sichuan has a high incidence of TB of approximately 100 cases per 100 000 people [7], with the second highest TB caseload among the provinces in China [8, 9]. Furthermore, the extensively drug-resistant (XDR)-TB notification rate significantly increased from 2010 to 2017 in Southwest China [10]. Approximately 9% of incident TB cases worldwide are among patients with HIV infection [11]. However, the characteristics of DR-TB isolates from patients with TB and HIV infection remain unclear in Sichuan. The GeneXpert assay is wildly applied for the rapid detection of TB in Sichuan. However, its detection rate and performance characteristics need to be updated.

In this study, we performed a comprehensive and updated retrospective investigation of the current drug resistance profiles of *M. tuberculosis* in the Sichuan core area from January 2018 to December 2020 and also analysed the local performance characteristics of the GeneXpert assay. This study is a continuation of our previous work [3].

## Methods

### Study setting

This study was conducted at the Chengdu Public Health Clinical Centre (https://www.phcc120.com/), which is the first and largest tertiary referral infectious disease specialist hospital, as well as the only officially designated medical institution for the treatment, research and prevention of TB in Sichuan. Overall, this hospital maintains the most comprehensive coverage of TB testing in China. This medical institution admits patients for TB hospital care, performs diagnostic evaluation of patients at high risk of MDR-TB, manages patients with various complications, and handles patients likely affected by TB. Moreover, this centre accepts TB samples from most hospitals, centres for disease control and prevention (CDCs), and health facilities in the province for drug susceptibility detection.

Patients who were diagnosed as being infected with TB [12] are admitted to the Jingju branch hospital of the Chengdu Public Health Clinical Centre. According to the requirements of the local health department, all TB strains isolated from TB patients in the core area of Sichuan should be sent to this hospital for drug susceptibility testing (DST), or patients should be directly transferred to this hospital for treatment and DST. Over 90% of TB cases in the core area of Sichuan are admitted here. Thus, this setting is a representative facility to reflect the status of TB drug resistance in Sichuan. The Clinical Laboratory of the Chengdu Public Health Clinical Centre has approximately 50 staff members and is divided into several professional groups, including a microbiology group, special mycobacterial group and molecular diagnostic group, with more than 300 tests on TB, AIDS and liver disease performed. This laboratory is also authorised to perform HIV screening and confirmation.

All admitted TB patients who stayed in the hospital while under treatment are required to receive HIV testing universally. Not all outpatient TB cases received HIV testing, because during 2018–2020, HIV testing is not mentioned as a necessary step for TB diagnosis at the country level [12]. According to the history of epidemiology, outpatients, who had multiple sex partners, history of intravenous drug use, or recurrent oral ulcers, etc., being suspected as HIV carriers, were subjected to HIV testing. The outpatient doctors also comprehensively judged whether patients with TB were at high risk of HIV infection, based on the symptoms, medical history and other related information. Testing was performed prior to TB diagnosis, with HIV infection confirmed by a combination of screening and antibody confirmation tests. The screening tests included enzyme-linked immunosorbent assay, a chemiluminescence assay, immunofluorescence assay and rapid test, and the antibody confirmation tests included western blotting, a line immunoassay and recombinant immunoblot assay. When screening and antibody confirmation tests did not confirm HIV in patients, PCR-based methods were applied [13, 14]. The patients with diagnosed HIV infection in this hospital could receive free antiretroviral therapy.

The routine detection procedure for TB was as follows. Patients suspected to have TB were diagnosed by hospital TB specialists, with two samples collected per patient. The samples were then subjected to *M. tuberculosis* culture (BD BACTEC MGIT 960 culture system; Sparks, MD, USA) and the GeneXpert (GeneXpert GX-XVI system; Cepheid, Sunnyvale, CA, USA) test, according to the manufacturers' instructions. The GeneXpert test was optional and was performed at the patient's expense. Drug sensitivity tests were conducted if the *M. tuberculosis* culture results were positive, regardless of the hospitalisation status of the patient. The treatment of MDR-TB was performed according to guidelines [15, 16].

Consecutive TB isolates were obtained from January 2018 to December 2020 and subjected to strain identification and DST. For patients with multiple isolates of one or more species, only the first isolate of each species was included in further analysis [17]. However, in patients with two TB isolates obtained from different samples, such as the sputum and bronchoscopy fluid, both isolates were subjected to DST. Non-tuberculous mycobacteria were excluded. TB isolates from patients with TB and HIV coinfection were included in the ‘TB_HIV group’. TB isolates from patients with negative HIV testing results and those at low risk of HIV infection being not subjected to HIV testing were included in the ‘general TB group’. The *M. tuberculosis* strains were stratified according to HIV infection status, patient age and calendar year of isolation for further analysis. Sample information, such as the patient's age, sex, HIV status, strain isolation year, type of the species and strain identification results, were obtained from the Laboratory Information System of the Chengdu Public Health Clinical Centre.

### *Mycobacterium tuberculosis* culture and drug sensitivity testing

The sputum, bronchoalveolar lavage fluid, pleural fluid, cerebrospinal fluid, ascites, pericardial effusion, gastric juice, secretions, pathological tissues, stools and urine were collected for mycobacterial culture. The confirmed *M. tuberculosis* isolates were then subjected to DST using DST kits purchased from Autobio Diagnostics Co., Ltd. (China Food and Drug Administration (CFDA) production licence 20160058; Zhengzhou, China) and Encode Medical Engineering Co., Ltd. (CFDA production licence 20010311; Zhuhai, China), according to the manufacturers' instructions. The Autobio DST kits were used from January 2018 to January 2019, and the Encode DST kits were used from January 2019 to December 2020. The result evaluation system for *para*-aminosalicylic acid (PAS) was updated in October 2019.

Briefly, the sensitivities of isolates to anti-TB drugs (ATDs) were determined using a microplate dilution method with 7H9 broth media [3]. The inoculum size was set at 200 μl per well, with low and high optimised concentrations of each drug determined by the manufacturer based on the Clinical and Laboratory Standards Institute (CLSI) guidelines [18]. After 7 days of culture, contamination of the culture medium was evaluated. If no contamination was observed, the reading was performed after 12 or 13 days of culture. A total of 11 anti-TB drugs were tested at low and medium concentrations in two wells. The four first-line drugs included isoniazid (INH; 0.20 and 0.80 μg/ml), rifampicin (RIF; 4.0 and 8.0 μg/ml), streptomycin (STR; 4.0 and 8.0 μg/ml) and ethambutol (EMB; 2.5 and 5.0 μg/ml). The seven second-line drugs included the fluoroquinolone drugs levofloxacin (LFX; 2.0 and 8.0 μg/ml) and moxifloxacin (MFX; 0.5 and 2.0 μg/ml); the oral bacteriostatic drug PAS (2.0 and 8.0 μg/ml) and oral rifabutin (RFB; 0.75 and 3.0 μg/ml); and the injectable drugs amikacin (AMK; 1.0 and 4.0 μg/ml), kanamycin (KM; 2.5 and 10.0 μg/ml) and capreomycin (CM; 2.5 and 10.0 μg/ml). The *M. tuberculosis* standard strain H37Rv (ATCC 27294), which is susceptible to all ATDs, was used as the control in each round of testing. Interlaboratory confirmation tests were conducted for quality control.

### Definitions of drug resistance

TB resistant to at least one of the four first-line and seven second-line drugs was defined as DR-TB. TB resistant to INH and RIF was defined as MDR-TB. TB resistant to INH, RIF, and either fluoroquinolones or a second-line anti-TB injectable drug (KM, CM or AMK), but not to both, was defined as pre-XDR-TB. TB resistant to at least INH, RIF, any member of the quinolone family, and at least one second-line anti-TB injectable drug was defined as XDR-TB [3, 19].

### GeneXpert MTB/RIF assay

The GeneXpert assay was conducted as previously described [3]. Briefly, 1 ml of each sample was added to 2 ml of the Xpert sample reagent, and the resulting solution was mixed for 1 min and digested at room temperature for 15 min with intermittent mixing. Subsequently, 2 ml of the sample solution was applied to the GeneXpert MTB/RIF GX-XVI system to determine the presence of *M. tuberculosis* and evaluate the resistance to RIF. Samples with unsuccessful GeneXpert results, including uncertain and failed results, were subjected to the second round of testing or excluded. The performance of the GeneXpert detection test was analysed for different samples (sputum, bronchoalveolar lavage fluid, pleural fluid, etc.). As the sample structure (proportion of different kinds of samples from 2018 to 2020) may have affected the sensitivity of the test, changes in the sample structure from 2018 to 2020 were further analysed.

### Statistical analysis

Data were analysed using SPSS Statistics (version 19.0; SPSS Inc., Chicago, IL, USA) and RStudio (1.3.1093). Continuous variables are presented as the median and interquartile range (IQR). Comparisons among groups and between pairs were performed using the Kruskal–Wallis test and Dunn's test, respectively. Categorical variables are presented as numbers and percentages and were analysed using the *χ*^2^ test or Fisher's exact test. The sensitivity (%), specificity (%), *κ* value, negative predictive value (NPV) and positive predictive value (PPV) of the GeneXpert assay were evaluated and compared with DST results for RIF [20]. Statistical significance was set at *P* < 0.05.

### Ethics statement

This study was approved by the ethics committee of the Chengdu Public Health Clinical Centre. Patient personal information was not included in this study. Because of the retrospective nature of the study, the requirement for written informed consent was waived. All methods were used in accordance with the relevant guidelines and regulations of the Declaration of Helsinki.

## Results

### Overall drug resistance patterns of TB isolates

From January 2018 to December 2020, a total of 10 946 culture-confirmed TB isolates were reported. Among these, 6749 isolates were from the outpatient department, 3197 isolates were from the inpatient department, 945 isolates were from 20 health facilities (16 isolates from district/county CDCs, three isolates from hospitals and one isolate from a third-party medical testing centre), and 55 isolates were of unknown origin. In total, 120 of these isolates were obtained from patients confirmed with HIV coinfections by laboratory tests. Approximately 28.7% (3107/10 826) isolates from the general TB group contained HIV testing results (Supplementary file 1). Furthermore, 7067 isolates and 3759 isolates of the general TB group were from men and women, respectively, and 102 isolates and 18 isolates of the TB_HIV group were from men and women, respectively. Additionally, the men having TB isolates were significantly older than the women having TB isolates (median age: 44.0 (IQR: 26.0–59.0) *vs.* 30.0 (IQR: 23.0–48.0), *P* *<* 0.001). The frequency of TB and HIV coinfection was also higher in the men having TB isolates than in the women having TB isolates (1.46% *vs.* 0.48%, *P* *<* 0.001).

Among the *M. tuberculosis* isolates, the order of resistance to the 11 ATDs (sorted from high to low) was as follows: INH (21.0%), RIF (14.2%), STR (13.6%), RFB (9.1%), PAS (8.6%), MFX (7.3%), LFX (5.8%), EMB (4.9%), KM (3.7%), CM (3.4%) and AMK (2.5%). The isolates with single-drug resistance, first-line ATD resistance, second-line ATD resistance, multidrug resistance, extensive drug resistance and universal drug resistance comprised 32.1%, 25.5%, 20.7%, 12.2%, 1.5% and 0.1% of the strain pool, respectively. No significant differences in drug resistance characteristics were observed between the general TB and TB_HIV groups (Fisher's exact test; Table 1).

**Table 1.** Drug resistance patterns of *Mycobacterium tuberculosis* isolates (*n* = 10 946)

Drug resistance	General TB (%(95%CI)) (*n* = 10 826)	TB_HIV (%(95%CI)) (*n* = 120)
INH	21.0%(20.2–21.8)	19.2%(12.1–26.2)
STR	13.6%(13.0–14.2)	10.8%(5.2–16.4)
RIF	14.2%(13.6–14.9)	10.8%(5.2–16.4)
EMB	4.9%(4.5–5.3)	6.7%(2.2–11.1)
RFB	9.1%(8.6–9.6)	7.5%(2.8–12.2)
AMK	2.5%(2.2–2.8)	3.3%(0.1–6.6)
CM	3.4%(3.1–3.8)	2.5%(0.0–5.3)
KM	3.7%(3.3–4.1)	5.0%(1.1–8.9)
LFX	5.8%(5.4–6.2)	3.3%(0.1–6.6)
MFX	7.3%(6.8–7.8)	5.8%(1.6–10.0)
PAS	8.6%(8.1–9.1)	8.3%(3.4–13.3)
Any drug resistance^a^	32.2%(31.3–33.1)	25.8%(18.0–33.7)
First-line drug resistance	25.5%(24.7–26.3)	22.5%(15.0–30.0)
Second-line drug resistance	20.8%(20.0–21.5)	16.7%(10.0–23.4)
Universal drug resistance^b^	0.1%(0.1–0.2)	0.0%(–)
MDR	12.3%(11.7–12.9)	9.2%(4.0–14.4)
Pre-XDR	4.0%(3.6–4.4)	2.5%(0.0–5.3)
XDR	1.5%(1.3–1.7)	1.7%(0.0–4.0)
INH + STR	10.9%(10.3–11.5)	9.2%(4.0–14.4)
INH + EMB	4.6%(4.2–5.0)	5.8%(1.6–10.0)
INH + RIF + STR	7.5%(7.0–8.0)	6.7%(2.2–11.1)
INH + RIF + EMB	3.7%(3.3–4.0)	4.2%(0.6–7.8)
INH + RIF + STR + EMB	2.7%(2.4–3.0)	2.5%(0.0–5.3)

TB, tuberculosis; HIV, human immunodeficiency virus; general TB, general tuberculosis patients; TB_HIV, tuberculosis patients with HIV; DST, drug sensitivity testing; 95% CI, 95% confidence interval; INH, isoniazid; STR, streptomycin; RIF, rifampicin; EMB, ethambutol; LFX, levofloxacin; AMK, amikacin; CM, capreomycin; MFX, moxifloxacin; PAS para-aminosalicylic acid; RFB, rifabutin; KM, kanamycin; MDR, multidrug-resistant; XDR, extensively drug-resistant.

a Resistant to at least one drug.

b Resistant to all 11 drugs involved in drug susceptibility testing.

### Sex-specific drug resistance profiles of TB isolates

Isolates from the male general TB patients, compared with those from the female patients, had significantly higher proportions of INH resistance (21.8% *vs.* 19.4%, *P* = 0.004), any drug resistance (33.1% *vs.* 30.2%, *P* = 0.002), first-line drug resistance (26.3% *vs.* 23.9%, *P* = 0.007) and universal drug resistance (0.2% *vs.* 0.0%, *P* = 0.027) (Fisher's exact test; Table 2). Comparison of the ATDs using the Kruskal–Wallis test showed no significant differences between the male and female groups.

**Table 2.** Sex-specific distribution of drug-resistant *Mycobacterium tuberculosis* isolates (*n* = 10 946)

Drug resistance	Male TB patients (%(95%CI)) (*n* = 7169)	Female TB patients (%(95%CI)) (*n* = 3777)
INH^a^	21.8%(20.8–22.7)	19.4%(18.2–20.7)
STR	13.9%(13.1–14.7)	13.0%(11.9–14.0)
RIF	14.5%(13.7–15.4)	13.6%(12.5–14.7)
EMB	5.1%(4.6–5.6)	4.6%(3.9–5.2)
RFB	9.4%(8.7–10.0)	8.6%(7.7–9.4)
AMK	2.5%(2.2–2.9)	2.4%(1.9–2.9)
CM	3.4%(3.0–3.8)	3.4%(2.8–4.0)
KM	4.0%(3.5–4.4)	3.3%(2.7–3.8)
LFX	5.9%(5.3–6.4)	5.5%(4.8–6.3)
MFX	7.5%(6.9–8.1)	7.0%(6.2–7.8)
PAS	8.8%(8.2–9.5)	8.2%(7.4–9.1)
Any drug resistance^a^^,^^b^	33.1%(32.0–34.2)	30.2%(28.7–31.7)
First-line drug resistance^a^	26.3%(25.3–27.3)	23.9%(22.5–25.3)
Second-line drug resistance	21.2%(20.3–22.2)	19.8%(18.5–21.0)
Universal drug resistance^a^^,^^c^	0.2%(0.1–0.3)	0.0%(0.0–0.1)
MDR	12.6%(11.8–13.4)	11.6%(10.5–12.6)
Pre-XDR	4.1%(3.6–4.5)	3.8%(3.2–4.4)
XDR	1.5%(1.2–1.7)	1.5%(1.1–1.9)
INH + STR	11.0%(10.3–11.8)	10.5%(9.5–11.5)
INH + EMB	4.8%(4.3–5.3)	4.3%(3.6–4.9)
INH + RIF + STR	7.8%(7.2–8.4)	7.0%(6.2–7.9)
INH + RIF + EMB	3.9%(3.5–4.4)	3.3%(2.7–3.8)
INH + RIF + STR + EMB	2.9%(2.5–3.3)	2.4%(1.9–2.9)

TB, tuberculosis; HIV, human immunodeficiency virus; DST, drug sensitivity testing; 95% CI, 95% confidence interval; INH, isoniazid; STR, streptomycin; RIF, rifampicin; EMB, ethambutol; LFX, levofloxacin; AMK, amikacin; CM, capreomycin; MFX, moxifloxacin; PAS para-aminosalicylic acid; RFB, rifabutin; KM, kanamycin; MDR, multidrug-resistant; XDR, extensively drug-resistant.

a Male TB patients *vs*. female TB patients, Fisher's exact test, INH: *P* = 0.004; any drug resistance, *P* = 0.002; first-line drug resistance, *P* = 0.007; universal drug resistance, *P* = 0.027.

b Resistant to at least one drug.

c Resistance to all 11 drugs involved in drug susceptibility testing.

### Age-specific drug resistance profiles of TB isolates

The isolates were divided into the following five groups based on the age of the patients: <15, 15–24, 25–44, 45–64 and ≥65 years [1, 3]. Among these groups, the overall percentages of resistance to the 11 drugs, sorted from high to low, were as follows: 45–64 years, 35.6% (95% CI 33.9–37.3); 25–44 years, 33.2% (95% CI 31.6–34.7); 15–24 years, 30.2% (95% CI 28.4–32.1); ≥65 years, 27.9% (95% CI 25.7–30.0); and <15 years, 22.5% (95% CI 17.7–27.4) (Table 3). Apart from the resistance to CM, universal drug resistance and extensive drug resistance, resistances to ATDs among the five age-related groups of the TB isolates were significantly different (Fisher exact test, *P* < 0.05, Table 3). Similar results were also observed by comparing the groups associated with 45–64 and <15-year-old patients, as well as those associated with 25–44 and <15-year-old patients. When the data for the seven second-line ATDs were grouped together for the Kruskal–Wallis test, the group associated with 45–64-year-old patients showed significantly higher resistance than that associated with <15-year-old patients (*S* = −16.143, *P* = 0.003, adjusted *P* = 0.032, Dunn's test). Notably, universal drug-resistant strains were isolated from all five subgroups (1, 1, 7, 4 and 2 isolates, respectively).

**Table 3.** Age-specific drug resistance characteristics of patients with tuberculosis (*n* = 10 946)

Drug resistance	Age (%(95%CI))					*P* values^a^
	<15 (*n* = 284)	15–24 (*n* = 2438)	25–44 (*n* = 3591)	45–64 (*n* = 2996)	≥65 (*n* = 1637)	
INH	13.4%(9.4–17.3)	19.4%(17.9–21.0)	23.2%(21.8–24.6)	23.4%(21.9–24.9)	15.2%(13.5–17.0)	<0.001
STR	6.0%(3.2–8.7)	13.4%(12.0–14.7)	15.2%(14.1–16.4)	15.0%(13.7–16.3)	8.9%(7.5–10.2)	<0.001
RIF	9.5%(6.1–12.9)	14.7%(13.3–16.1)	16.7%(15.5–17.9)	15.2%(13.9–16.5)	7.0%(5.8–8.3)	<0.001
EMB	2.5%(0.7–4.3)	5.2%(4.3–6.0)	5.9%(5.1–6.7)	5.2%(4.4–6.0)	2.4%(1.6–3.1)	<0.001
RFB	5.6%(2.9–8.3)	10.0%(8.8–11.2)	10.4%(9.4–11.4)	9.9%(8.8–11.0)	3.9%(3.0–4.8)	<0.001
AMK	0.4%(0.0–1.0)	2.1%(1.5–2.7)	2.6%(2.1–3.2)	3.1%(2.5–3.8)	1.8%(1.2–2.5)	= 0.002
CM	1.4%(0.0–2.8)	3.2%(2.5–3.9)	3.5%(2.9–4.1)	3.5%(2.9–4.2)	3.7%(2.8–4.6)	= 0.333
KM	1.8%(0.2–3.3)	2.2%(1.6–2.8)	3.2%(2.7–3.8)	5.1%(4.3–5.9)	4.9%(3.8–5.9)	<0.001
LFX	1.4%(0.0–2.8)	4.3%(3.5–5.1)	6.5%(5.7–7.3)	6.6%(5.8–7.5)	5.6%(4.4–6.7)	<0.001
MFX	1.4%(0.0–2.8)	5.5%(4.6–6.4)	8.4%(7.5–9.3)	8.6%(7.6–9.6)	6.3%(5.1–7.5)	<0.001
PAS	3.5%(1.4–5.7)	7.8%(6.8–8.9)	8.8%(7.8–9.7)	9.6%(8.6–10.7)	8.5%(7.1–9.8)	= 0.002
Any drug resistance^b^	22.5%(17.7–27.4)	30.2%(28.4–32.1)	33.2%(31.6–34.7)	35.6%(33.9–37.3)	27.9%(25.7–30.0)	<0.001
First-line drug resistance	18.7%(14.1–23.2)	24.3%(22.6–26.0)	27.7%(26.2–29.1)	28.3%(26.7–29.9)	18.4%(16.5–20.3)	<0.001
Second-line drug resistance	10.9%(7.3–14.5)	19.6%(18.1–21.2)	21.7%(20.3–23.0)	23.2%(21.7–24.7)	17.4%(15.6–19.2)	<0.001
Universal drug resistance^c^	0.4%(0.0–1.0)	0.0%(0.0–0.1)	0.2%(0.1–0.3)	0.1%(0.0–0.3)	0.1%(0.0–0.3)	= 0.332
MDR	6.0%(3.2–8.7)	12.7%(11.4–14.0)	14.7%(13.5–15.9)	13.0%(11.8–14.2)	5.9%(4.8–7.1)	<0.001
Pre-XDR	0.0%(–)	4.3%(3.5–5.1)	4.5%(3.8–5.2)	4.7%(4.0–5.5)	1.6%(1.0–2.3)	<0.001
XDR	0.4%(0.0–1.0)	0.8%(0.4–1.1)	2.1%(1.7–2.6)	1.7%(1.2–2.1)	0.9%(0.5–1.4)	<0.001
INH + STR	4.2%(1.9–6.6)	10.4%(9.2–11.6)	12.5%(11.4–13.6)	12.2%(11.0–13.4)	6.6%(5.4–7.8)	<0.001
INH + EMB	2.1%(0.4–3.8)	4.8%(4.0–5.6)	5.6%(4.9–6.4)	4.8%(4.0–5.6)	2.2%(1.5–2.9)	<0.001
INH + RIF + STR	1.4%(0.0–2.8)	7.7%(6.7–8.8)	9.1%(8.1–10.0)	8.2%(7.2–9.2)	3.7%(2.8–4.6)	<0.001
INH + RIF + EMB	1.1%(0.0–2.2)	3.7%(3.0–4.5)	4.7%(4.0–5.4)	3.9%(3.2–4.6)	1.4%(0.8–2.0)	<0.001
INH + RIF + STR + EMB	0.4%(0.0–1.0)	2.5%(1.9–3.2)	3.6%(3.0–4.2)	2.9%(2.3–3.5)	1.2%(0.7–1.8)	<0.001

TB, tuberculosis; HIV, human immunodeficiency virus; DST, drug sensitivity testing; 95% CI, 95% confidence interval; INH, isoniazid; STR, streptomycin; RIF, rifampicin; EMB, ethambutol; LFX, levofloxacin; AMK, amikacin; CM, capreomycin; MFX, moxifloxacin; PAS para-aminosalicylic acid; RFB, rifabutin; KM, kanamycin; MDR-TB, multidrug-resistant tuberculosis; XDR, extensively drug-resistant.

a Fisher exact tests among five groups.

b Resistant to at least one drug.

c Resistance to all 11 drugs involved in drug susceptibility testing.

### Year-specific drug resistance profiles of TB isolates

The isolates were divided into three groups according to the year of isolation. Among the groups, the percentages of drug resistance to the 11 drugs, sorted from high to low, were as follows: 2018 > 2019 > 2020 (Table 4). Apart from universal drug resistance, the resistances to ATDs were significantly different among the three groups (Fisher's exact test, *P* < 0.05, Table 4). Compared with the 2020 group, the 2018 and 2019 groups had higher percentages of resistance to RIF (12.4% *vs.* 15.7%, *P* < 0.001; 12.4% *vs.* 14.8%, *P* = 0.002, respectively), AMK (1.5% *vs.* 3.6%, *P* < 0.001; 1.5% *vs.* 2.6%, *P* = 0.001, respectively), CM (1.5% *vs.* 3.1%, *P* < 0.001; 1.5% *vs.* 5.4%, *P* < 0.001, respectively), MFX (5.1% *vs.* 11.0%, *P* < 0.001; 5.1% *vs.* 6.7%, *P* = 0.002, respectively), any drugs (26.2% *vs.* 31.1%, *P* < 0.001; 26.2% *vs.* 38.2%, *P* < 0.001, respectively), any second-line drugs (13.5% *vs.* 19.2%, *P* < 0.001; 13.5% *vs.* 28.3%, *P* = 0.002, respectively) and multidrugs (10.6% *vs.* 14.3%, *P* < 0.001; 10.6% *vs.* 12.2%, *P* = 0.029, respectively). By contrast, the 2020 group had relatively lower percentages of XDR isolates than those in the 2018 and 2019 groups (0.8% *vs.* 2.7%, *P* = 0.007; 0.8% *vs.* 1.1%, *P* = 0.216, respectively).

**Table 4.** Distribution of drug-resistant *Mycobacterium tuberculosis* isolates obtained in different years (*n* = 10 946)

Drug resistance	2018 (%(95%CI)) (*n* = 2989)	2019 (%(95%CI)) (*n* = 4184)	2020 (%(95%CI)) (*n* = 3773)	*P* values^a^
INH	23.9%(22.4–25.5)	20.4%(19.2–21.6)	19.3%(18.0–20.6)	<0.001
STR	15.9%(14.6–17.2)	12.1%(11.1–13.1)	13.3%(12.2–14.4)	<0.001
RIF	15.7%(14.4–17.0)	14.8%(13.7–15.9)	12.4%(11.3–13.4)	<0.001
EMB	10.7%(9.6–11.8)	2.0%(1.6–2.4)	3.6%(3.0–4.1)	<0.001
RFB	13.0%(11.8–14.2)	8.1%(7.3–8.9)	7.1%(6.3–7.9)	<0.001
AMK	3.6%(2.9–4.2)	2.6%(2.1–3.0)	1.5%(1.1–1.9)	<0.001
CM	3.1%(2.5–3.7)	5.4%(4.7–6.1)	1.5%(1.1–1.9)	<0.001
KM	1.7%(1.2–2.1)	6.5%(5.7–7.2)	2.3%(1.8–2.8)	<0.001
LFX	9.3%(8.3–10.3)	4.8%(4.2–5.5)	4.0%(3.4–4.6)	<0.001
MFX	11.0%(9.9–12.1)	6.7%(5.9–7.4)	5.1%(4.4–5.8)	<0.001
PAS	3.2%(2.5–3.8)	15.9%(14.8–17.0)	4.9%(4.2–5.5)	<0.001
Any drug resistance^b^	31.1%(29.4–32.7)	38.2%(36.7–39.6)	26.2%(24.8–27.6)	<0.001
First-line drug resistance	27.9%(26.3–29.5)	25.1%(23.8–26.4)	23.9%(22.5–25.3)	= 0.001
Second-line drug resistance	19.2%(17.8–20.6)	28.3%(26.9–29.7)	13.5%(12.5–14.6)	<0.001
Universal drug resistance^c^	0.2%(0.0–0.4)	0.0%(0.0–0.1)	0.2%(0.0–0.3)	= 0.109
MDR	14.3%(13.1–15.6)	12.2%(11.2–13.2)	10.6%(9.6–11.6)	<0.001
Pre-XDR	5.8%(5.0–6.6)	3.6%(3.0–4.1)	3.0%(2.4–3.5)	<0.001
XDR	2.7%(2.2–3.3)	1.1%(0.8–1.5)	0.8%(0.6–1.1)	<0.001
INH + STR	13.4%(12.2–14.7)	9.8%(8.9–10.7)	10.0%(9.1–11.0)	<0.001
INH + EMB	9.9%(8.9–11.0)	1.9%(1.5–2.3)	3.4%(2.9–4.0)	<0.001
INH + RIF + STR	9.2%(8.2–10.2)	6.7%(5.9–7.4)	7.1%(6.3–8.0)	<0.001
INH + RIF + EMB	8.1%(7.1–9.1)	1.3%(1.0–1.7)	2.8%(2.3–3.3)	<0.001
INH + RIF + STR + EMB	6.2%(5.3–7.0)	0.7%(0.4–0.9)	2.2%(1.8–2.7)	<0.001

TB, tuberculosis; HIV, human immunodeficiency virus; DST, drug sensitivity testing; 95% CI, 95% confidence intervals; INH, isoniazid; STR, streptomycin; RIF, rifampicin; EMB, ethambutol; LFX, levofloxacin; AMK, amikacin; CM, capreomycin; MFX, moxifloxacin; PAS para-aminosalicylic acid; RFB, rifabutin; KM, kanamycin; MDR, multidrug-resistant; XDR, extensively drug-resistant.

a Fisher exact tests among five groups.

b Resistant to at least one drug.

c Resistant to all 11 drugs involved in drug susceptibility testing.

### Performance characteristics of the GeneXpert assay

From 2018 to 2020, 7586 samples were subjected to GeneXpert detection and drug sensitivity testing, of which 2486 (five uncertain results and three subjected to the second round of testing with successful results), 2797 (13 uncertain results and two subjected to the second round of testing with successful results) and 2303 (21 uncertain results and five subjected to the second round of testing with successful results) samples were tested in 2018, 2019 and 2020, respectively. The 10 samples with uncertain results that were subjected to the second round of testing were included in further data analysis, while the 29 samples that were not subjected to the second round of testing were excluded. No significant differences in the success rates were observed between sputum and other samples (99.45% (6100/6134) *vs.* 99.66% (1447/1452), *P* > 0.05).

Approximately 69.0% (7557/10 946) of the isolates had both DST and successful GeneXpert assay results. For RIF resistance testing of the *M. tuberculosis* isolates, we observed a sensitivity of 79.0% (95% CI 76.6–81.4), specificity of 95.9% (95% CI 95.5–96.4), PPV of 76.7 (95% CI 74.2–79.2), NPV of 96.4 (95% CI 96.0–96.9) and *κ* value of 0.741 (95% CI 0.73–0.752). Similar results were obtained for the general TB group (Table 5). The sensitivity and specificity of the GeneXpert test for the strains from the TB_HIV group were 80.4% (95% CI 78.0–82.9) and 96.2% (95% CI 95.7–96.7), respectively (Table 5). The PPV, NPV and *κ* values for the TB_HIV group were 78.0% (95% CI 75.5–80.6), 96.7% (95% CI 96.2–97.1) and 0.584 (95% CI 0.451–0.717), respectively (Table 5). The sensitivities for the three consecutive year groups (2018, 2019 and 2020) demonstrated a downward trend (80.9 (95% CI 76.8–85.0) *vs.* 80.2 (95% CI 76.4–84.1) *vs.* 75.4 (95% CI 70.7–80.2), respectively; Table S1).

**Table 5.** Performance characteristics of the GeneXpert assay compared to drug susceptibility testing for rifampicin between the general TB and TB_HIV groups (*n* = 7557)

Xpert MTB/RIF	DST-RIF		Total	PPV (%(95%CI))	NPV (%(95%CI))
	resistant	sensitive			
2018–2020, general TB (*n* = 7470)					
GX-RIF(+)	851	256	1107	76.8(74.3–79.3)	
GX-RIF(−)	223	6140	6363		96.4(96.0–96.9)
Total	1074	6396	7470		
Sensitivity(%(95%CI))	79.2(76.8–81.6)				
Specificity(%(95%CI))		95.9(95.5–96.4)			
*κ*(%(95%CI))			0.743(0.732–0.754)		
2018–2020, TB_HIV (*n* = 87)					
GX-RIF(+)	7	4	11	78.0(75.5–80.6)	
GX-RIF(−)	4	72	76		96.7(96.2–97.1)
Total	11	76	87		
Sensitivity(%(95%CI))	80.4(78.0–82.9)				
Specificity(%(95%CI))		96.2(95.7–96.7)			
*κ*(%(95%CI))			0.584(0.451–0.717)		

DST, drug susceptibility testing; MTB, *Mycobacterium tuberculosis*; GX, GeneXpert; RIF, rifampicin; 95% CI, 95% confidence interval; NPV, negative predictive value; PPV, positive predictive value.

Regarding the GeneXpert sensitivity of different samples, the highest sensitivity (84.2%) was obtained with bronchoalveolar lavage and pleural fluids, the second highest sensitivity (79.0%) was obtained with sputum samples, and the lowest sensitivity (65.5%) was obtained with the other samples (Table S2). Additionally, the GeneXpert test showed statistically different sensitivity results for the three types of samples (*χ*^2^ = 9.045, *P* = 0.011). Furthermore, no significant changes in the sample structure were observed among the different year groups (Table S3).

## Discussion

This study revealed the drug resistance profiles of *M. tuberculosis* isolates from patients with TB in Sichuan, China, from January 2018 to December 2020. Generally, the DR-TB status improved annually. The overall proportion of DR-TB among culture-confirmed TB was 32.1% (3514/10 946). This result was similar to the proportion reported in a previous study conducted in Chinese Jiangxi (33.1% (352/1063)) [21] and Chinese Dalian (31.1% (1106/3552)) [22], but higher than those reported for Central China (14.8% (531/3580)), Eastern China (12.8% (1889/14 757)) and Western China (10.8% (1198/11 097)) [23] and lower than that found in our previous study, which was conducted from 2014 to 2017 (52.7% (3933/7470)) [3]. Similar results were also obtained for MDR-TB. The overall proportion of MDR-TB among culture-confirmed TB was 12.2% (1340/10 946), which was similar to those in Chinese Jiangxi (14.8% (157/1063)) [21], Eastern China (12.8% (2967/32 707)) [23] and Western China (12.3% (1957/15 929)) [23], as well as in our previous 2014–2017 study (14.6% (1088/7470)) [3]. However, the proportion was higher than those in Chinese Dalian (10.1% (359/3552)) [22], Central China (8.5% (501/5899)). By contrast, the overall proportion of XDR-TB in this study was 1.5% (162/10 946), which was similar to those in Jiangxi (2.4% (26/1063)) [21], Dalian (2.1% (75/3552)) [22] and Sichuan from 2014 to 2017 (1.4% (103/7470)) [3]. These findings indicated that while the DR-TB status in Sichuan remains serious, the situation has improved.

Meanwhile, in our study, approximately 1.1–3.7% (120/10 826 to 120/3227) of the incident TB cases were among patients with HIV infection, which was notably lower than the worldwide proportion of 9% [11], the Mainland China proportion of 7.4% [24] and the China proportion of 7.3% released by WHO [25, 26]. The potential reasons for the discrepancies between our reported number and that at the country level as presented by other reports [24–26] might be because of the different data sources. Some data are from routine surveillance of TB. While, some data sources are from non-routine surveillance of HIV prevalence in TB patients [26]. And due to the retrospective nature, we could not possibly better explain those reasons. Indeed, in our study, some TB_HIV patients could be missed because of the low proportion of HIV testing for TB patients. The extended unavailability of HIV testing for the general TB group might be due to the HIV testing being optional for TB patients. WHO has recommended HIV testing for all TB patients for a long time [27]. Our tertiary referral infectious disease hospitals and related health facilities could consider a full HIV screening coverage strategy for all TB patients according to the requirement of WHO. It could not only help to find more TB-HIV patients for early disease control but also decrease the discrepancies of TB-HIV incidence at the country level from different reports [24–26]. Our work brings attention to the prevalence of MDR-, XDR- and universal DR-TB infections, as well as to the low proportion of HIV testing for outpatient TB cases in Southwest China.

With respect to the age, the 45–64- and 25–44-year-old groups had the highest proportions of MDR-TB, which were significantly higher than that in the <15-year-old group (14.7% and 13.0% *vs.* 6.0%, respectively). In addition, the 25–44-year-old group had a significantly higher XDR-TB proportion than those in the <15-year-old (2.1% *vs.* 0.4%) and 15–24-year-old (2.1% *vs.* 0.8%) groups. These results indicate that the patients with TB in the 25–64 years of age group, which consists of the majority of the labour force, are still at high risk for the development of MDR- and XDR-TB. Recent global estimates also show a considerable TB burden among young adults, which leads to unique challenges in effective diagnosis and treatment [28]. Meanwhile, it has been reported that the 25–44 years of age group is the most affected group, with the highest incidence rate of RIF-resistant TB [29]. Thus, more attention should be focused on this group of patients.

Regarding the different study years, the overall TB drug resistance status gradually improved from 2018 to 2020. Our results demonstrated a continuous improvement in overall control of TB drug resistance in Sichuan over the past 3 years, a finding consistent with that of a previous study [3]. Currently, TB treatment in China is reimbursable through medical insurance, with a basic medical insurance coverage rate of 96.8% [30]. Meanwhile, support from overseas groups, such as the Bill & Melinda Gates Foundation, aids in the development of programmes to strengthen TB control [31], which may have contributed to the observed annual improvement of the TB drug resistance status.

The MDR- and XDR-TB cases decreased annually from 2018 to 2020 (MDR-TB incidences in 2018, 2019 and 2020: 14.3%, 12.2% and 10.6%, respectively; XDR-TB incidences in 2018, 2019 and 2020: 2.7%, 1.1% and 0.8%, respectively; Table 4). While, the rate of resistance to PAS peaked in 2019 (2018, 2019 and 2020: 3.2%, 15.9% and 4.9%, respectively; Table 4). The fluctuation in the PAS resistance rate may have been due to the replacement of the detection reagents in 2019. Although the DST result interpretation standards of the old (Autobio) and the new (Encode) kits were the same, variations still occurred at the low concentration (2.00 μg/ml PAS). Moreover, the examiners in 2019 may have been stricter on DST result interpretation of the PAS resistance. Upon discovery of this peak, the manufacturers updated the result evaluation system, with PAS resistance rates returning to the normal range (<5%) by 2020.

In the first quarter of 2020, the COVID-19 pandemic positively and negatively influenced TB control. On the one hand, the prevention and control measures enacted against severe acute respiratory syndrome coronavirus 2 (SARS-CoV-2), such as wearing facial masks, reduced the incidence of TB infections. On the other hand, the Chengdu Public Health Clinical Centre, which previously mainly performed TB diagnosis and treatment, was also designated as a hospital for COVID-19 diagnosis and treatment [32]. Thus, some suspected TB patients might have been reluctant to visit hospitals because of the fear of COVID-19 infection. However, COVID-19 may not have significantly impacted the admission of patients with TB to the hospital. It is believed that the disease burden of TB will decrease as long as COVID-19 can be successfully controlled [33].

In this study, we found an increase in the use of GeneXpert among TB cases with DST, from 10.9% (812/7470) [3] in 2014–2017 to 69.0% (7557/10 946) in 2018–2020. This rapid detection method is highly accepted by hospitals and patients with TB. In a recent systematic review, it has been reported that the pooled sensitivity and specificity of Xpert for RIF-resistant TB detection in China were 92% and 98%, respectively [34]. However, recent data from Sichuan Province were not included in this systematic review. Furthermore, only one study on TB in Sichuan was conducted with a relatively small sample size (*n* = 268) of isolates from January 2008 to May 2010 [35]. Our findings demonstrated a lower sensitivity but steadier specificity (81.1% and 94.6%, for 2014–2017 [3] and 79.5% and 96.0%, for 2018–2020) than the findings of the above-mentioned systematic review [34]. The observed sensitivities in our studies were comparable to those in the United States (81.0%) [36] and Morocco (78.8%) [37].

The sensitivities of the GeneXpert assay were not significantly different in 2018, 2019 and 2020 (80.9%, 80.2% and 75.4%, respectively; Table S1). However, the sensitivities showed a downward trend during the three consecutive years. Since the Xpert buffer was designed for sputum samples, application of this method to other specimens may have affected the outcome by increasing false-negative results and decreasing overall sensitivity [38]. In our study, no significant difference in the success rate between sputum and other samples (99.45% *vs.* 99.66%) was observed. Meanwhile, we found that GeneXpert was the most sensitive with bronchoalveolar lavage and pleural fluids (mainly pulmonary TB) and the least sensitive with other samples (mainly extrapulmonary TB; Table S2). Similar results have also been reported in a recent multicentre study [38]. Regarding the effect of the sample structure on GeneXpert performance, no significant changes in the sample structure were observed among the different years. However, the proportions of bronchoalveolar lavage and pleural fluid samples, for which the test showed the highest sensitivity, gradually decreased from 14.95% in 2018 to 13.20% in 2020. The proportions of other samples also decreased from 5.90% in 2018 to 4.80% in 2020. These data suggest that the sample structure has little effect on the sensitivity of GeneXpert. Thus, we speculated that some TB cases might be caused by new mutants, which are not recognised by the five rpoB molecular beacons used in the GeneXpert assay [39]. The new and inexpensive GeneXpert MTB/RIF Ultra assay and gene sequencing-based detection may be highly accurate potential options [36, 40]. In addition, guaranteeing the quality of sample collection and preparation for the GeneXpert test may aid in maximising its performance for MDR-TB control. For example, strict collection of morning sputum samples and avoidance of saliva may increase the positivity rate of GeneXpert testing.

The limitations of our study are as follows. First, the information about new and previously treated TB cases, medication details and drug side effects was not available. Second, HIV testing was not universally available for all outpatient TB cases, 71.3% of the individuals in the general TB group did not have HIV testing results, thus potentially leading to misclassification of patients in the general TB group. Third, we did not collect TB data from all CDCs, hospitals and healthcare facilities in Sichuan. Fourth, not all types of ATDs were included, and not all isolates were submitted to GeneXpert testing. These limitations might have resulted in an inaccurate evaluation of the overall TB status in Sichuan Province. Thus, more comprehensive collection of information from databases of patients with TB from multiple centres is required for future studies.

## Conclusion

This study summarises the status of ATD resistance in patients with TB in Sichuan, China, from January 2018 to December 2020. Our findings indicate a decreasing trend in overall ATD resistance of TB isolates from 2018 to 2020. No significant differences were observed in drug resistance characteristics between the general TB and TB_HIV groups. However, MDR-TB remains severe, especially among middle-aged patients. The increased usage rate of GeneXpert, and guaranteeing the quality of sample collection and preparation for GeneXpert testing may aid in maximising the test performance for MDR-TB control. However, the gradual decrease in the sensitivity of the GeneXpert assay should be noticed. And HIV testing for each outpatient TB case is recommended for early treatment and management.

## Data Availability

The data that support the findings of this study are openly available. Please find the datasets in Supplementary file 1.

## Supplementary material

For supplementary material accompanying this paper visit http://doi.org/10.1017/S0950268822000127. click here to view supplementary material
