# Supplementary material for: Drug resistance characteristics of Mycobacterium tuberculosis isolates obtained between 2018 and 2020 in Sichuan, China
Source: Epidemiol Infect. 2022 Jan 28;150:e27. doi: 10.1017/S0950268822000127 (PMC8888273; doi:10.1017/S0950268822000127)
Supplement: Supplementary file 1 [file hygsup.zip › S0950268822000127sup002.docx]

**Table S1. Performance characteristics of the GeneXpert assay compared to drug susceptibility testing for rifampicin (2018 vs. 2019 vs. 2020, n=7,557).**

| **Xpert MTB/RIF** | **DST-RIF** | | **Total** | **PPV [%(95%CI)]** | **NPV [%(95%CI)]** |
| --- | --- | --- | --- | --- | --- |
|  | resistant | sensitive |  |  |  |
| **2018 (n=2,287)** | | | | | |
| **GX-RIF(+)** | 284 | 83 | 367 | 77.3(73.1–81.6) |  |
| **GX-RIF(-)** | 67 | 1853 | 1920 |  | 96.5(95.6–97.3) |
| **Total** | 351 | 1936 | 2287 |  |  |
| **Sensitivity[%(95%CI)]** | 80.9(76.8–85.0) |  |  |  |  |
| **Specificity[%(95%CI)]** |  | 95.7(94.8–96.6) |  |  |  |
| **Kappa[%(95%CI)]** |  |  | 0.752(0.733–0.771) |  |  |
| **2019(n=2,786)** | | | | | |
| **GX-RIF(+)** | 334 | 90 | 424 | 78.7(74.8–82.6) |  |
| **GX-RIF(-)** | 82 | 2280 | 2362 |  | 96.5(95.7–97.2) |
| **Total** | 416 | 2370 | 2786 |  |  |
| **Sensitivity[%(95%CI)]** | 80.2(76.4–84.1) |  |  |  |  |
| **Specificity[%(95%CI)]** |  | 96.2(95.4–96.9) |  |  |  |
| **Kappa[%(95%CI)]** |  |  | 0.759(0.741–0.777) |  |  |
| **2020 (n=2,484)** | | | | | |
| **GX-RIF(+)** | 240 | 87 | 327 | 73.3(68.6–78.1) |  |
| **GX-RIF(-)** | 78 | 2079 | 2157 |  | 96.3(95.5–97.1) |
| **Total** | 318 | 2166 | 2484 |  |  |
| **Sensitivity[%(95%CI)]** | 75.4(70.7–80.2) |  |  |  |  |
| **Specificity[%(95%CI)]** |  | 95.9(95.1–96.8) |  |  |  |
| **Kappa[%(95%CI)]** |  |  | 0.706(0.684–0.728) |  |  |

DST, drug susceptibility testing; MTB, *Mycobacterium tuberculosis*; GX, GeneXpert; RIF, rifampicin; 95%CI, 95% confidence interval; NPV, negative predictive value; PPV, positive predictive value.
