# Supplementary material for: Drug resistance characteristics of Mycobacterium tuberculosis isolates obtained between 2018 and 2020 in Sichuan, China
Source: Epidemiol Infect. 2022 Jan 28;150:e27. doi: 10.1017/S0950268822000127 (PMC8888273; doi:10.1017/S0950268822000127)
Supplement: Supplementary file 1 [file hygsup.zip › S0950268822000127sup003.docx]

**Table S2. Performance characteristics of the GeneXpert assay compared to drug susceptibility testing for rifampicin (different sample types, n=7,557).**

| Xpert MTB/RIF | DST-RIF | | Total | PPV [%(95%CI)] | NPV [%(95%CI)] |
| --- | --- | --- | --- | --- | --- |
|  | resistant | sensitive |  |  |  |
| Sputum (n=6,108) | | | | | |
| GX-RIF(+) | 686 | 216 | 902 | 76.0(73.2–78.8) |  |
| GX-RIF(-) | 182 | 5024 | 5206 |  | 96.5(96.0–97.0) |
| Total | 868 | 5240 | 6108 |  |  |
| Sensitivity[%(95%CI)] | 79.0(76.3–81.7) |  |  |  |  |
| Specificity[%(95%CI)] |  | 95.8(95.3–96.4) |  |  |  |
| Kappa[%(95%CI)] |  |  | 0.737(0.724–0.75) |  |  |
| BALF & PF (n=1,065) | | | | | |
| GX-RIF(+) | 134 | 33 | 167 | 80.2(74.2–86.2) |  |
| GX-RIF(-) | 25 | 873 | 898 |  | 97.2(96.1–98.2) |
| Total | 159 | 906 | 1065 |  |  |
| Sensitivity[%(95%CI)] | 84.2(78.6–89.9) |  |  |  |  |
| Specificity[%(95%CI)] |  | 96.3(95.1–97.5) |  |  |  |
| Kappa[%(95%CI)] |  |  | 0.79(0.763–0.817) |  |  |
| Other samples (n=384) | | | | | |
| GX-RIF(+) | 38 | 11 | 49 | 77.5(65.8–89.2) |  |
| GX-RIF(-) | 20 | 315 | 335 |  | 94.0(91.4–96.5) |
| Total | 58 | 326 | 384 |  |  |
| Sensitivity[%(95%CI)] | 65.5(53.2–77.7) |  |  |  |  |
| Specificity[%(95%CI)] |  | 96.6(94.6–98.5) |  |  |  |
| Kappa[%(95%CI)] |  |  | 0.758(0.734–0.782) |  |  |

DST, drug susceptibility testing; MTB, *Mycobacterium tuberculosis*; GX, GeneXpert; RIF, rifampicin; 95%CI, 95% confidence interval; NPV, negative predictive value; PPV, positive predictive value. BALF, bronchoalveolar lavage fluid; PF, pleural fluid. Other samples: cerebrospinal fluid, ascites, pericardial effusion, gastric juice, secretions, pathological tissues, stools, and urine
