# Supplementary material for: Drug resistance characteristics of Mycobacterium tuberculosis isolates obtained between 2018 and 2020 in Sichuan, China
Source: Epidemiol Infect. 2022 Jan 28;150:e27. doi: 10.1017/S0950268822000127 (PMC8888273; doi:10.1017/S0950268822000127)
Supplement: Supplementary file 1 [file hygsup.zip › S0950268822000127sup004.docx]

**Table S3. Sample structure changes in GeneXpert from 2018 to 2020 (n=7,557).**

| **sample types/year** | **2018** | **2019** | **2020** | **R×C Chi-square value** | ***P*-value** |
| --- | --- | --- | --- | --- | --- |
| **Sputum** | 1,810(79.14) | 2,261(81.15) | 2,037(82.00) | 8.271 | 0.082 |
| **BALF & PF** | 342(14.95) | 395(14.17) | 328(13.20) |  |  |
| **Other samples** | 135(5.902) | 130(4.666) | 119(4.790) |  |  |
| **Total** | 2,287 | 2,786 | 2,484 |  |  |

BALF, bronchoalveolar lavage fluid; PF, pleural fluid. Other samples: cerebrospinal fluid, ascites, pericardial effusion, gastric juice, secretions, pathological tissues, stools, and urine
